# Supplementary material for: VARUS: sampling complementary RNA reads from the sequence read archive
Source: BMC Bioinformatics. 2019 Nov 8;20:558. doi: 10.1186/s12859-019-3182-x (PMC6842140; doi:10.1186/s12859-019-3182-x)
Supplement: Supplementary file 1 — Additional file 1 The supplementary material includes command lines and additional figures. [file 12859_2019_3182_MOESM1_ESM.pdf]

VARUS: Sampling Complementary RNA Reads from the Sequence Read Archive
Supplementary Materials

Mario Stanke[1,2], Willy Bruhn[1], Felix Becker[1,2], and Katharina J. Hoff[1,2]

[1]Institute for Mathematics and Computer Science, University of Greifswald, Walther-Rathenau-Str. 47, 17489, Greifswald, Germany
[2]Center for Functional Genomics of Microbes, University of Greifswald, Felix-Hausdorff-Str. 8, 17489, Greifswald, Germany

April 13, 2019

Contents

1 Supplementary Methods **1**
1.1 Assembly & Reference Annotation Processing 1
1.2 Running VARUS 2
1.3 Downloading Manually Selected RNA-Seq Libraries 3
1.4 Aligning Manually Selected RNA-Seq Libraries 3
1.5 Running BRAKER 3

2 Supplementary Figures **3**

3 Supplementary Tables **5**

1 Supplementary Methods

1.1 Assembly & Reference Annotation Processing

The names of genome FASTA file entries downloaded from NCBI are long and complex. For our purposes, unique sequence IDs in the header are sufficient. We trimmed FASTA headers and replaced dots in sequence names by underscores:

```
cat original.fa | perl -pe 's/(>\S*)\.(\d+)\s.*/$1_$2/g;' > genome.fa
```

Dots in sequence names were replaced by underscores in the corresponding annotation files:

```
cat original.gff | perl -pe 's/\./_/' > annot.gff3
```

BRAKER is by design unable to predict genes with frameshift errors or genes that have parts located on both strands. Reference annotation files were checked for genes with such issues using GenomeTools [Gremme et al., 2013]:

```
gt  gff3  -force  -tidy  -o annot_tidy_by_GenomeTools.gff3  \
  -retainids  -sort  annot.gff3  2>  errors_by_GenomeTools
cat errors_by_GenomeTools | grep -v gbunit | cut -f6 -d' ' | sort | uniq -c | wc -l
```

Reference annotation files in GFF3 format were converted to GTF format using GenomeTools (this also removed numerous file entries that are not related to the structures of protein coding genes, e.g. rRNA features, etc.):

```
gt  gff3_to_gtf  -force  -o annot_by_GenomeTools.gtf annot_tidy_by_GenomeTools.gff3
```

Finally, GTF format files were run through `validate_gtf.pl` from the Eval package [Keibler and Brent, 2003]:

```
validate_gtf.pl -f annot_by_GenomeTools.gtf
```

The resulting file `annot_by_GenomeTools.fixed.gtf` was used for measuring gene prediction accuracy of BRAKER.

1.2 Running VARUS

VARUS was run with the command

```
runVARUS.pl --readFromTable species.txt --aligner=HISAT > runVARUS.log 2> runVARUS.err
```

Here, `species.txt` is a tab or semicolon separated file with the binomial names and paths to the genome files:

```
Anopheles gambiae          Anopheles_gambiae.fa
Bombus terrestris          Bombus_terrestris.fa
Chlamydomonas reinhardtii  Chlamydomonas_reinhardtii.fa
Cucumis sativus            Cucumis_sativus.fa
Medicago truncatula        Medicago_truncatula.fa
Drosophila melanogaster    Drosophila_melanogaster.fa
Fragaria vesca             Fragaria_vesca.fa
Parasteatoda tepidariorum  Parasteatoda_tepidariorum.fa
Prunus persica             Prunus_persica.fa
Hymenolepis microstoma     Hymenolepis_microstoma.fa
Saccharomyces cerevisiae   Saccharomyces_cerevisiae.fa
Verticillium dahliae       Verticillium_dahliae.fa
```

In the same directory was a text file `VARUSparameters.txt` with the following content. `runVARUS.pl` reads its parameters from there:

```
--batchSize 50000
--maxBatches 1000
--blockSize 5000
--cost 0.001
--deleteLater 0
--estimator 2
--exportObservationsToFile 1
--exportParametersToFile 1
--fastqDumpCall fastq-dump
--genomeDir ./genome/
--lambda 10.0
--loadAllOnce 0
--mergeThreshold 10
--outFileNamePrefix ./
--pathToParameters ./VARUSparameters.txt
--pathToRuns ./
--pathToVARUS /home/mario/VARUS/Implementation/
--qualityThreshold 5
--runThreadN 8
--verbosityDebug 1
```

Individual runs, e.g. to repeat a single experiment were run like this:

```
runVARUS.pl --aligner=HISAT --runThreadN=4 --readFromTable=0 --createindex=1 --verbosity=5 \
  --latinGenus=Prunus --latinSpecies=persica --speciesGenome=Prunus_persica.fa \
  --logfile=Prunus_persica.log 2> Prunus_persica.err
```

1.3 Downloading Manually Selected RNA-Seq Libraries

Manually selected RNA-Seq libraries were downloaded using SRA-Toolkit [Leinonen et al., 2010] as follows ($i is the library ID):

```
fastq-dump $i
```

The argument `--split-files` was appended for paired libraries.

1.4 Aligning Manually Selected RNA-Seq Libraries

All RNA-Seq libraries were aligned using HISAT2 [Kim et al., 2015] version 2.1.0. A genome index was built with `hisat2-build`:

```
hisat2-build genome.fa genome
```

Unpaired RNA-Seq libraries were aligned as follows (${i} is the library ID):

```
hisat2 -p 15 -x genome -U ${i}.fastq -S ${i}.sam 1> ${i}.hisat2.out
```

Paired RNA-Seq libraries were aligned with:

```
hisat2 -p 15 -x genome -1 ${i}_1.fastq -2 ${i}_2.fastq -S ${i}.sam 1> ${i}.hisat2.out
```

SAM format files were subsequently converted to BAM format and sorted by target sequence name using Samtools [Li et al., 2009] version 1.8-20-g4ff8062:

```
samtools view -@18 -bSh ${i}.sam -o ${i}.bam
samtools sort -@18 -n ${i}.bam -o ${i}.s.bam
```

Introns were extracted from BAM files using the AUGUSTUS auxprogs tool `bam2hints` [Hoff and Stanke, 2019]:

```
bam2hints --intronsonly --in=${i}.s.bam --out=${i}.introns
```

Introns from different libraries for the same species were concatenated into a single file per species.

1.5 Running BRAKER

BRAKER [Hoff et al., 2015] version 2.1.3 was executed in order to annotate genomes in a fully automated fashion on the basis of VARUS RNA-Seq alignments and alignments of manually selected RNA-Seq libraries with GeneMark-ES/ET [Lomsadze et al., 2014] version 4.38 and AUGUSTUS [Stanke et al., 2008] version 3.3.2 (intron.hints are either intron hints from manually selected RNA-Seq libaries or sampled by VARUS):

```
braker.pl --genome=genome.fa --hints=hints.introns --softmasking \
  --cores=8 --eval=annot_by_GenomeTools.fixed.gtf
```

The command line option `--fungus` was appended for *Saccharomyces cerevisiae* and *Verticillium dahliae.*

2 Supplementary Figures

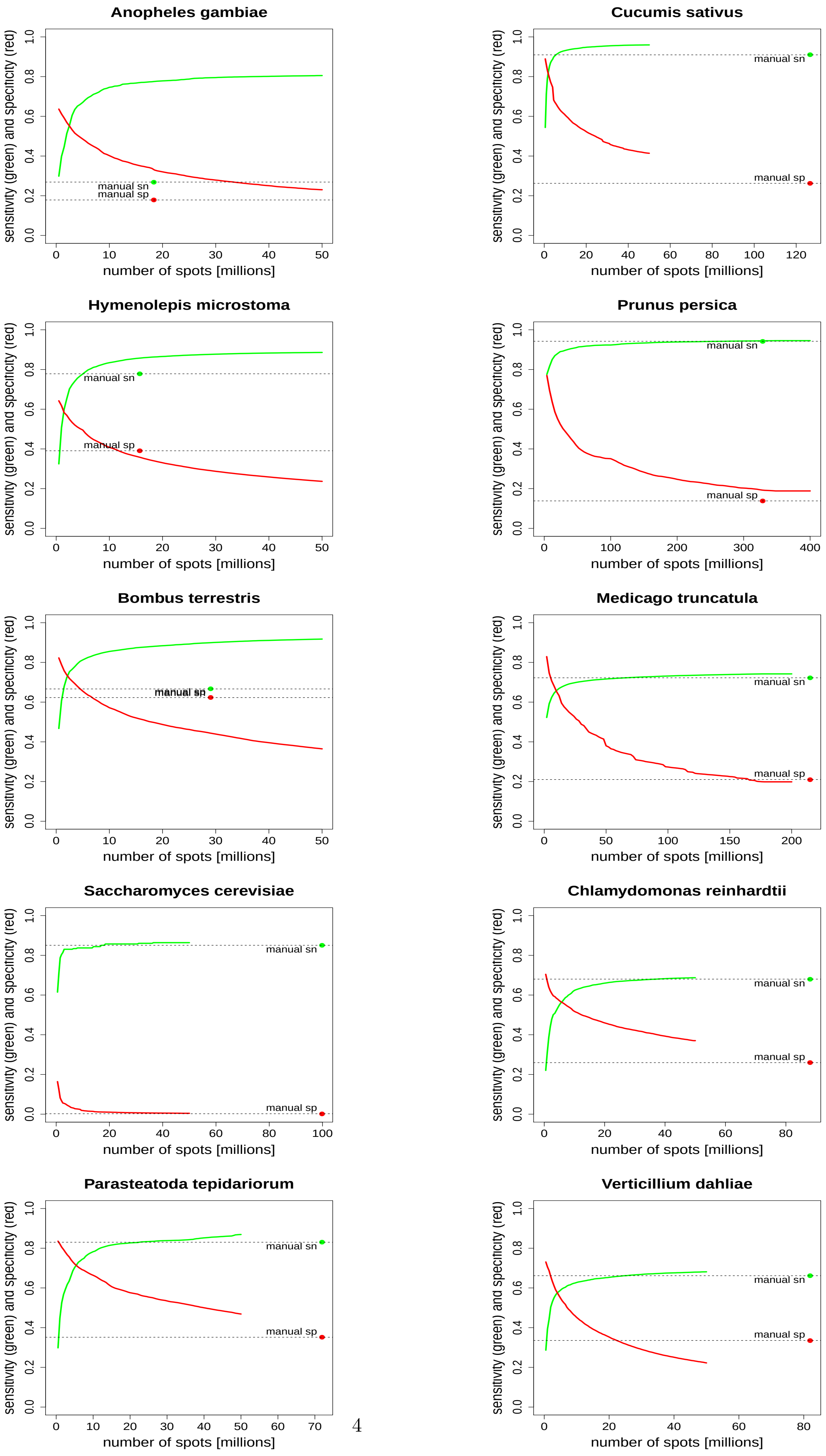

Supplementary Figure 1: Intron Accuracy. See Figure 3 of main text for a description.

3 Supplementary Tables

Species	Assembly version	Genome size (bp)
Anopheles gambiae	AgamP3	265027044
Bombus terrestris	Bter_1.0	248654244
Cannabis sativa	ASM186575v1	585823666
Chlamydomonas reinhardtii	v3.0	120404952
Cucumis sativus	ASM407v2	195669205
Drosophila melanogaster	r6.07	133880608
Fragaria vesca	FraVesHawaii_1.0	214373013
Hymenolepis microstoma	HMIC002	182136974
Medicago truncatula	MedtrA17_4.0	412924424
Parasteatoda tepidariorum	Ptep_2.0	1445396121
Prunus persica	NCBIv2	227569171
Saccharomyces cerevisiae	R64	12071326
Verticillium dahliae	ASM15067v2	33900324

Supplementary Table 1: Species names, assembly versions and genome sizes of species used for testing VARUS with BRAKER.

Species	Annotation source
Anopheles gambiae	ftp://ftp.ncbi.nlm.nih.gov/genomes/all/GCF/000/005/575/GCF_000005575.2_AgamP3/GCF_000005575.2_AgamP3_genomic.gff.gz
Bombus terrestris	ftp://ftp.ncbi.nlm.nih.gov/genomes/all/GCF/000/214/255/GCF_000214255.1_Bter_1.0/GCF_000214255.1_Bter_1.0_genomic.gff.gz
Cannabis sativa	–
Chlamydomonas reinhardtii	ftp://ftp.ncbi.nlm.nih.gov/genomes/all/GCF/000/002/595/GCF_000002595.1_v3.0/GCF_000002595.1_v3.0_genomic.gff.gz
Cucumis sativus	ftp://ftp.ncbi.nlm.nih.gov/genomes/all/GCF/000/004/075/GCF_000004075.2_ASM407v2/GCF_000004075.2_ASM407v2_genomic.gff.gz
Drosophila melanogaster	ftp://ftp.flybase.net/genomes/Drosophila_melanogaster/dmel_r6.07_FB2015_04/gff/dmel-all-r6.07.gff.gz
Fragaria vesca	ftp://ftp.ncbi.nlm.nih.gov/genomes/all/GCF/000/184/155/GCF_000184155.1_FraVesHawaii_1.0/GCF_000184155.1_FraVesHawaii_1.0_genomic.gff.gz
Hymenolepis microstoma	ftp://ftp.ncbi.nlm.nih.gov/genomes/all/GCA/000/469/805/GCA_000469805.2_HMIC002/GCA_000469805.2_HMIC002_genomic.gff.gz
Medicago truncatula	ftp://ftp.ncbi.nlm.nih.gov/genomes/all/GCF/000/219/495/GCF_000219495.3_MedtrA17_4.0/GCF_000219495.3_MedtrA17_4.0_genomic.gff.gz
Parasteatoda tepidariorum	ftp://ftp.ncbi.nlm.nih.gov/genomes/all/GCF/000/365/465/GCF_000365465.2_Ptep_2.0/GCF_000365465.2_Ptep_2.0_genomic.gff.gz
Prunus persica	ftp://ftp.ncbi.nlm.nih.gov/genomes/all/GCF/000/346/465/GCF_000346465.2_Prunus_persica_NCBIv2/GCF_000346465.2_Prunus_persica_NCBIv2_genomic.gff.gz
Saccharomyces cerevisiae	ftp://ftp.ncbi.nlm.nih.gov/genomes/all/GCF/000/146/045/GCF_000146045.2_R64/GCF_000146045.2_R64_genomic.gff.gz
Verticillium dahliae	ftp://ftp.ncbi.nlm.nih.gov/genomes/all/GCF/000/150/675/GCF_000150675.1_ASM15067v2/GCF_000150675.1_ASM15067v2_genomic.gff.gz

Supplementary Table 2: Sources of reference annotations that were used for assessing gene prediction accuracy of BRAKER1.

Species	RNA-Seq libraries
Anopheles gambiae	ERR2192559, SRR5145619, SRR8249138, SRR8249143, SRR8249144
Bombus terrestris	ERR047506, SRR098297, SRR5614828
Chlamydomonas reinhardtii	DRR059691, SRR057479, SRR094743, SRR6505183, SRR6870054
Cucumis sativus	SRR1197971, SRR3872505, SRR517595, SRR7609888, SRR3872503, SRR3872508, SRR6895195
Drosophila melanogaster	SRR023505, SRR023546, SRR023608, SRR026433, SRR027108
Fragaria vesca	SRR5217589, SRR5217590, SRR5217591, SRR5217592, SRR6320486, SRR6320488, SRR6320491
Hymenolepis microstoma	ERR337976
Medicago truncatula	ERR1830535, SRR3726837, SRR3938252, SRR7175037, SRR7473103, SRR7772259
Parasteatoda tepidariorum	DRR046998, DRR047007, DRR047017, DRR054577, SRR6941355, SRR6941356
Prunus persica	SRR5274660, SRR6001701, SRR6001786, SRR6374787, SRR7262551, SRR7469172
Saccharomyces cerevisiae	SRR7448271, SRR7696788, SRR7696806, SRR7774370, SRR8261652
Verticillium dahliae	SRR2087157, SRR2087158, SRR6130340

Supplementary Table 3: Manually selected RNA-Seq libraries.

Species	Exon Sens.		Exon Spec.	
	VARUS	Manual	VARUS	Manual
Anopheles gambiae	**64.7**	55.2	54.5	**56.8**
Bombus terrestris	**78.2**	71.7	**70.9**	67.6
Chlamydomonas reinhardtii	**62.1**	61.6	50.0	**50.1**
Cucumis sativus	**86.7**	86.0	65.3	**68.7**
Drosophila melanogaster	**78.6**	75.9	80.2	80.2
Fragaria vesca	**84.9**	83.6	62.0	**66.1**
Hymenolepis microstoma	**80.3**	77.9	59.5	**62.8**
Medicago truncatula	**67.4**	67.1	62.0	62.0
Parasteatoda tepidariorum	**81.9**	80.4	**63.6**	62.8
Prunus persica	85.1	85.1	62.2	**62.9**
Saccharomyces cerevisiae	**72.5**	71.2	**87.4**	84.8
Verticillium dahliae	**64.2**	63.2	**64.8**	64.6

Supplementary Table 4: Exon level gene prediction accuracy of AUGUSTUS (with hints) trained on the basis of GeneMark-ET predictions within BRAKER1 measured with respect to existing reference annotations. Please note that not all reference annotations are of high quality.

References

[Gremme et al., 2013] Gremme, G., Steinbiss, S., and Kurtz, S. (2013). Genometools: a comprehensive software library for efficient processing of structured genome annotations. *IEEE/ACM Transactions on Computational Biology and Bioinformatics (TCBB)*, 10(3):645–656.

[Hoff et al., 2015] Hoff, K., Lange, S., Lomsadze, A., Borodovsky, M., and Stanke, M. (2015). BRAKER1: unsupervised RNA-Seq-based genome annotation with GeneMark-ET and AUGUSTUS. *Bioinformatics*, 32(5):767–769.

[Hoff and Stanke, 2019] Hoff, K. J. and Stanke, M. (2019). Predicting genes in single genomes with augustus. *Current protocols in bioinformatics*, 65(1):e57.

[Keibler and Brent, 2003] Keibler, E. and Brent, M. R. (2003). Eval: a software package for analysis of genome annotations. *BMC bioinformatics*, 4(1):50.

[Kim et al., 2015] Kim, D., Langmead, B., and Salzberg, S. L. (2015). Hisat: a fast spliced aligner with low memory requirements. *Nature methods*, 12(4):357.

[Leinonen et al., 2010] Leinonen, R., Sugawara, H., Shumway, M., and Collaboration, I. N. S. D. (2010). The sequence read archive. *Nucleic acids research*, 39(suppl_1):D19–D21.

[Li et al., 2009] Li, H., Handsaker, B., Wysoker, A., Fennell, T., Ruan, J., Homer, N., Marth, G., Abecasis, G., and Durbin, R. (2009). The sequence alignment/map format and SAMtools. *Bioinformatics*, 25(16):2078–2079.

[Lomsadze et al., 2014] Lomsadze, A., Burns, P., and Borodovsky, M. (2014). Integration of mapped RNA-Seq reads into automatic training of eukaryotic gene finding algorithm. *Nucleic Acids Research*, 42(15):e119.

[Stanke et al., 2008] Stanke, M., Diekhans, M., Baertsch, R., and Haussler, D. (2008). Using native and syntenically mapped cdna alignments to improve de novo gene finding. *Bioinformatics*, 24(5):637.
